# Medical Chemistry for the Use of Students and the Profession; Being a Manual of the Science, with Its Applications to Toxicology, Physiology, Therapeutics, Hygiene, &c.

**Published:** 1848-08

**Authors:** 


*Medical Chemistry for the use of Students and the Profession; being a Manual of the Science, with its applications to Toxicology, Physiology, Therapeutics, Hygiene, &c.* By D. P. Gardner, M. D., Formerly Professor of Chemistry in the Philadelphia College, and of Chemistry and Natural Philosophy in Hampden Sydney College, Va.; Corresponding Member of the Lyceum of Natural History, New York. Small 8vo. pp. 396. Philadelphia. Lea & Blanchard: 1848.

We are now well supplied with "Manuals" of Chemistry; and so long as useful novelties are produced, we are neither disposed to cavil, nor to enquire too closely whether they be indispensable or advisable. Every such production must contain something valuable; and it must be borne in mind that they are not written for the expert, but for those who are desirous of being made so.

The work before us differs more perhaps in title than in execution from the ordinary manuals of chemistry—for it must of necessity treat of the fundamental phenomena, and, to a certain extent, of the laws of such phenomena. Accordingly, after an Introduction, in which the properties of matter and specific gravity are investigated, we have Part I., which considers the "Chemical Forces"—under the heads of heat, light, electricity, galvanism, animal galvanism and affinity. Part II. follows, which investigates "the simple bodies of greatest importance." Part III. treats of "organic chemistry," from which—and we cannot see the reason—the author separates Part IV., which is devoted to "Animal Chemistry." Part V. considers the metals, with the exception of arsenic and antimony, which are classed with the simple bodies in Part II. We do not think the classification a happy one: but this is of minor importance.

The author has long studied and taught the department of science on which he now writes, and is doubtless familiar with it. We have no question, therefore, that his work may be regarded as an accurate exposition of most of the topics which it embraces. We have not had opportunity, however, to examine closely into this matter; and have turned mainly to one or two points of interest connected with the application of chemistry to medicine.

It may be recollected, that some years ago Dr. Gardner published an article in the "American Journal of the Medical Sciences," in which he suggested, that sulphuretted hydrogen might be an active ingredient in malaria. Since that time accurate observation has exploded the notion. We were, therefore, curious to know what are his present views.

"*Malaria.*—The Italian writers have for some time suspected a connection between the presence of sulphur, and the poisonous atmosphere (*malaria*) which produces bilious remittent and intermittent fevers. The late Professor Daniell found sulphuretted hydrogen in the waters taken from the most sickly portions of the African coast" [Dr. Gardner has omitted to state, that the gas was not discoverable in the waters when taken from the river, but was the product of after decomposition] "and Dr. Marcet in those from the Chinese coast, which is equally unhealthy. I have succeeded in detecting the presence of this body, both in the air and waters of marshes and shallow streams in Virginia, and in locations where bilious remittent fever was active. (See the American Journal of the Medical Sciences, January, 1842.) I do not, however, agree with Prof. Daniell and others, that sulphuretted hydrogen is the malarious body, but believe, that sulphur is a component of a certain unstable organic body, which constitutes the poison. This organic body may be obtained by precipitating the vapour from a marsh, in the morning before sunrise, and it blackens sulphuric acid. It consists of sulphur, carbon, hydrogen, and probably of oxygen. It readily decomposes, emitting a putrid odour, and appears to be decomposed by the rays of the sun. It is destroyed by fire, does not rise high in the air, is decomposed by plants, which appear to remove its water, an essential ingredient of the body.

"This substance entering the lungs, is probably decomposed into sulphuretted hydrogen, carbonic acid and water. Hence" [?] "the sulphuretted hydrogen is most probably the noxious body; this enters the circulation, affects the blood, produces nervous symptoms, which have the intermittent or remittent type, affects the liver, and, if taken in large quantity, produces nervous and vascular prostration, with all the symptoms of yellow fever [!]. These would be the effects of sulphuretted hydrogen [!!]. The best remedies, calomel, and, in chronic cases, nitro-muriatic acid, both contain chlorine. The first of these is decomposed in the body, and they both evolve chlorine, which, as we have seen, neutralizes the poisonous properties of sulphuretted hydrogen."

Yet all this is the merest hypothesis, possessing none of the exactness that ought to appertain to a chemical investigation. Independently of all idea of the agency of sulphuretted hydrogen in the production of periodical fevers having been abandoned by almost all its supporters, and considered to rest on scarcely a particle of foundation; the author appears to be of opinion, that intermittent and remittent and yellow fevers are all producible and produced by the same malarious emanation, in favour of which view we know of neither facts nor arguments!

We do not think that physiology has engaged as much of the author's attention as chemistry. So at least we should judge from the evidences afforded in the volume before us. For example, after all the information obtained, from time to time, of the value of the saliva in digestion, he seems to have made up his mind that it is essential to human digestion, on the strength of a single case.

"The absolute necessity of the saliva has been occasionally proved in cases where the secretion was diminished or destroyed from disease of the glands. Professor Pattison saw a striking instance of this fact, in the case of a man who had cut his throat, and in whom the wound remained open for a long time. Nutritious food was administered by the severed orifice of the œsophagus, but the man rapidly wasted, and was on the eve of death from emaciation. It was observed, that whenever his meals were presented, an abundant discharge of saliva took place and ran out of his mouth, for he had lost the power of swallowing. The secretion had been formerly thrown away, but in consequence of his emaciated condition, attention was turned to it, and the Professor suggested that it should be collected, and introduced along with the food into his stomach. From this time the man improved, and finally recovered his health. *From this striking case it will appear that the saliva is essential to digestion in man.*" p. 318.

Dr. Gardner *decides*, with Blondlot, whom he repeatedly calls *Blondot*, and Berard and Barreswil, that the acid reaction of the gastric juice depends upon the presence of the acid phosphate of soda or lime, and free lactic acid. "Neither free hydrochloric nor acetic acids [acid] exist [exists] in the stomach." Not a word is said of the experiments made in this country on the only case perhaps in which the gastric secretions of man have been obtained in the best state for examination—we allude to that of San Martin, the Canadian. In those secretions free muriatic acid always seemed to exist.

On the subject of endosmose and exosmose we expected more detail than Dr. Gardner has given. He passes by without notice the recent researches of Matteucci, in the interesting little volume reprinted in this country, which led to the conclusions in relation to the agency of the septum, which we gave in the "Examiner" for February last, (p. 114.)

The very recent work of Liebig on the motion of the juices doubtless had not reached the author when he penned his observations on this topic.

On the whole, perhaps, the greatest objection to Dr. Gardner's physiological inferences is the hasty deduction from chemical premises by no means established, yet enunciated by him with all the confidence which the exact sciences alone authorize. This has been a common error of the chemists, and it applies strongly to Liebig himself, who, in many instances, has arrived too speedily at his deductions; and has thus tended to diminish the confidence of the unreflecting in a branch of science important above all others in enabling us to unravel the phenomena of living bodies, inasmuch as there is probably not a change nor motion—the result of living action—that is not chemical in its nature.

We observe typographical errors, which the author will have, we hope, an opportunity of correcting in a future edition. Proper names are, at times, inaccurately given. Blondlot, as we have seen; and we notice Majendie and Despetz, for Magendie and Despretz.

